# Relationship between innate immunity, soluble markers and metabolic-clinical parameters in HIV+ patients ART treated with HIV-RNA<50 cp/mL

**DOI:** 10.7448/IAS.17.4.19718

**Published:** 2014-11-02

**Authors:** Chiara Dentone, Daniela Fenoglio, Alessio Signori, Giovanni Cenderello, Alessia Parodi, Federica Bozzano, Michele Guerra, Pasqualina De Leo, Valentina Bartolacci, Eugenio Mantia, Giancarlo Orofino, Francesca Kalli, Francesco Marras, Paolo Fraccaro, Mauro Giacomini, Giovanni Cassola, Bianca Bruzzone, Giuseppe Ferrea, Claudio Viscoli, Gilberto Filaci, Andrea De Maria, Antonio Di Biagio

**Affiliations:** 1 Infectious Diseases, Sanremo Hospital, Sanremo, Italy; 2 Center of Excellence for Biomedical Research, University of Genoa, Sanremo, Italy; 3 DIMI, Center of Excellence for Biomedical Research, University of Genoa, Genoa, Italy; 4 Section of Biostatistics, University of Genoa, Genoa, Italy; 5 Infectious Diseases, Galliera Hospital, Genoa, Italy; 6 Center of Excellence for Biomedical Research, University of Genoa, Genoa, Italy; 7 DIMES, Center of Excellence for Biomedical Research, University of Genoa, Genoa, Italy; 8 Infectious Diseases, La Spezia Hospital, La Spezia, Italy; 9 Infectious Diseases, Savona Hospital, Savona, Italy; 10 Infectious Diseases, Albenga Hospital, Albenga, Italy; 11 Infectious Diseases, Alessandria Hospital, Alessandria, Italy; 12 Infectious Diseases, Amedeo di Savoia Hospital, Torino, Italy; 13 Immunology, Giannina Gaslini Institute, Genoa, Italy; 14 DIBRIS, University of Genoa, Genoa, Italy; 15 DIBRIS, Center of Excellence for Biomedical Research, University of Genoa, Genoa, Italy; 16 UO Hygiene, San Martino Hospital IRCCS, Genoa, Italy; 17 Infectious Diseases, San Martino Hospital IRCCS, Genoa, Italy; 18 University of Genoa, Genoa, Italy; 19 DIMI, University of Genoa, Genoa, Italy; 20 Center of Excellence for Biomedical Research, San Martino Hospital, Genoa, Italy; 21 Infectious Diseases, San Martino Hospital IRCCS, Genoa, Italy; 22 Center of Excellence for Biomedical Research, University of Genoa, Genoa, Italy

## Abstract

**Introduction:**

The persistence of immune activation and inflammation in HIV patients with HIV-RNA (VL) undetectable causes many co-morbidities [1–3]. The aim of this study is to correlate monocytes (m) and NK cell activation levels, soluble markers and oxidative stress with clinical, biochemical and metabolic data in HIV-1 infected patients with VL≤50 copies (cp)/mL on antiretroviral therapy.

**Materials and Methods:**

Multicentre, cross-sectional study in patients with VL≤50 cp/mL and on antiretroviral therapy by at least six months. We studied: activation/homing markers (CD38, HLA-DR, CCR-2, PDL-1) on inflammatory, intermediate, proinflammatory m; activatory receptors NKp30, NKp46 and HLA-DR on NK cells; soluble inflammatory (sCD14, adiponectina, MCP-1) and stress oxidative markers (dRoms, antiRoms). Univariate analyses are performed with non-parametric and Pearson tests. The significant correlations were adjusted for possible known confounding factors (smoking, Cytomegalovirus IgG serology, Raltegravir, Protease Inhibitor [PI] therapy and HCV-RNA) with multivariate analysis.

**Results:**

In the 68 patients the positive correlation between age and antiRoms was significant also after adjustment for PI use (p=0.05). The% CD8+T was associated with% proinflammatory m (p=0.043) and with their expression of CCR2 mean fluorescence intensity (MFI) (p=0.012). The% NKp46+ was positively correlated with CD4+T count (p=0.001). The fibrinogen was positively associated with dRoms (p=0.052) and the positive correlation between triglycerides and antiRoms has been confirmed (p<0.001); the impact of antiRoms on HDL/triglycerides ratio (p=0.006) was observed after adjustment for PI use. The BMI was associated with smoking (p=0.011). Only the maraviroc-treated patients showed minimal arterial pressure, fibrinogen and antiRoms lower (p=0.001, 0.004 e 0.006) and sCD14 values higher (p=0.029).

**Conclusions:**

Patients with long history of HIV infection and stable immunological and virological status showed interactions between acquired and innate immunity activation; moreover, the levels of some metabolic and inflammatory parameters correlate with oxidative stress values and innate immunity activation. The age, BMI and smoking impact metabolic and immunological parameters. The correlations between antiretroviral drugs and clinical-immunological parameters need further confirmations.

**Table 1:** Patients' characteristics

Age, years (median, IQR)	49 (46–54)
Sex, n males (%)	46 (68)
Prior AIDS events, n (%)	25 (37)
Co-infection HCV and/or HBV, n (%)	21 (31)
Current smoking, n (%)	42 (62)
BMI (median, IQR)	23.5 (20.6–25.5)
Nadir TCD4 (median, IQR)	202 (67–316)
Time since HIV+ diagnosis, years (median, IQR)	19 (16–22)
Time on antiretroviral therapy, years (median, IQR)	15 (9–16)
CD4+T at enrolment (median, IQR)	488 (370–607)
Antiretroviral therapy at enrolment, n (%)	PI 37 (54), RAL 39 (39), MVC 43 (63), NNRTI 26 (38)
